# VIEWPOINT FROM THE DIVISION OF BEHAVIORAL AND SOCIAL RESEARCH

**DOI:** 10.1093/geroni/igz038.1641

**Published:** 2019-11-08

**Authors:** John Haaga

**Affiliations:** National Institute on Aging, National Institutes of Health, Bethesda, Maryland, United States

## Abstract

Dr. Haaga will review accomplishments of the Division of Behavioral and Social Research and make projections for the future.

